# Near-complete genome sequence of *Mycoplasma meleagridis* isolated from a New Zealand turkey

**DOI:** 10.1128/mra.01077-25

**Published:** 2026-02-04

**Authors:** Andrew Wilson, Jonathan Foxwell, Ruy Jauregui, Yee Syuen Low, Ning Yuan, Harry Taylor, Mark Eames, Bede Busby, Michelle McCulley, Joseph O'Keefe

**Affiliations:** 1 Diagnostics, Readiness and Surveillance, Biosecurity New Zealand, Ministry for Primary Industries 206260 , Upper Hutt, New Zealand; Nanchang University, Nanchang, Jiangxi, China

**Keywords:** *Mycoplasma meleagridis*, New Zealand

## Abstract

We report a near-complete genome sequence of *Mycoplasma meleagridis* isolated from a turkey in New Zealand. Using long-read sequencing approaches, a single circular scaffold was assembled, and then annotated. The genome reported here is the first genetic characterization of *M. meleagridis* in New Zealand.

## ANNOUNCEMENT

In October 2024, *Mycoplasma meleagridis* infection was detected in a backyard flock of turkeys (*Meleagris gallopavo*) from the Northland region of New Zealand during an exotic disease incursion investigation into a respiratory disease outbreak (1). *M. meleagridis* is a host-specific pathogen of concern internationally for turkey husbandry (2). Evidence of *M. meleagridis* in New Zealand has, until present, solely relied on serological detection, and with no detection since 1997, the species has been considered exotic (3). This announcement describes the first bacterial isolation and genome characterization of a New Zealand strain.

*M. meleagridis* was initially detected by PCR assays as described below. The flock was resampled on 26 November 2024 to attempt bacterial isolation. Screening was performed on oropharyngeal and cloacal swabs from seven birds using a qPCR adapted from reference 4. Positives were confirmed by sequencing products of a generic 16S rDNA PCR adapted from reference 5 on a MinION flow cell (Oxford Nanopore Technologies [ONT], methods below). BLASTn analysis indicated 99.86% identity to strain NCTC-10153 (GenBank ID LR215042). Positive detections were from cloacal swabs, with three of seven birds positive for *M. meleagridis*.

*M. meleagridis* was isolated using ATCC medium: 243 HI Plus Medium with YE #3, liquid broth, and agar variants. Swabs were inoculated in 2 mL broth and directly onto solid agar, and all media were incubated at 37°C with 5% CO_2_ for 7 days. At scheduled time points (1 and 48 h) and at *ad-hoc* points in case of media pH change, whole broths were filtered (0.45 µM), and 10 µL sub-cultured to solid agar in triplicate. Agar was inspected for colonies with characteristic morphology as described in reference 6. Suspect colonies were excised into liquid broth and incubated for 96 h; DNA was extracted; and they were screened using the abovementioned qPCR. A representative isolate (W24_02583-18) derived from a cloacal swab of one turkey isolated 12 December 2024 was extracted on a larger scale (10 mL culture) for sequencing. Extractions were performed using the QIAamp DNA Mini Kit tissue protocol (Qiagen)—broths were centrifuged at 10,000 rcf for 2 min to pellet bacteria before the addition of lysis buffer. DNA concentration was assessed using the Qubit 1× dsDNA high-sensitivity assay (Thermo Fisher Scientific).

Libraries for sequencing were prepared using the Rapid Sequencing Kit V14 (SQK-RAD114, ONT) and sequenced on a MinION Mk1B with a R10.4.1 flow cell (ONT). Basecalling was performed with Dorado v7.4.14 using the high-accuracy model v4.3.0. Reads were trimmed with BBDuk v38.84 (7), then assembled using Flye v2.9.5 (8). After assembly, one circular scaffold was generated. Circularity was estimated automatically using Flye. The genome was annotated using National Center for Biotechnology Information’s Prokaryotic Genome Annotation Pipeline (PGAP) v6.9 (9). Completeness and contamination were assessed using CheckM v1.2.3. Genome assembly, annotation statistics, and CheckM results are presented in Table 1.

**TABLE 1:** Genome assembly and annotation statistics for the *Mycoplasma meleagridis* genome

	Statistic
Assembly feature	
Raw read number	355,303
Raw read N50	2.82 Kb
Trimmed read number	355,155
Trimmed read N50	2.82 Kb
Total genome size (bp)	639,447
Total GC content	26.1%
Number of scaffolds	1
Final genome coverage	700×
Completeness	97.69%
Contamination	<0.01%
Annotation features	
CDSs	518
rRNAs	2, 2, 2 (5S, 16S, 23S)
tRNAs	33
ncRNAs	3
Pseudogenes	29
Total # of genes annotated	589

All laboratory protocols detailed above were performed according to the manufacturer’s instructions. For software, default parameters were used.

In summary, we report the first near-complete genome sequence of *M. meleagridis* isolated from a New Zealand turkey. The availability of this genome provides a valuable reference for future surveillance, diagnostic assay development, and comparative studies.

## Data Availability

Raw sequence data have been submitted to NCBI’s Sequence Read Archive (SRA) under BioProject accession no. PRJNA1211651. The genome reported here has been deposited in GenBank under accession no. CP182213.
